# Processing moving visual scenes during upright stance in elderly patients with mild cognitive impairment

**DOI:** 10.7717/peerj.10363

**Published:** 2020-11-18

**Authors:** Martin Kucharik, Zuzana Kosutzka, Jozef Pucik, Michal Hajduk, Marian Saling

**Affiliations:** 1 Centre of Experimental Medicine, Slovak Academy of Sciences, Bratislava, Slovakia; 2 Second Department of Neurology, Faculty of Medicine, Comenius University, Bratislava, Slovakia; 3 Institute of Electronics and Photonics, Faculty of Electrical Engineering and Information Technology, Slovak University of Technology in Bratislava, Bratislava, Slovak Republic; 4 Department of Psychology, Faculty of Arts, Comenius University, Bratislava, Slovakia; 5 Department of Psychiatry, Faculty of Medicine, Comenius University, Bratislava, Slovakia; 6 Center for Psychiatric Disorders Research—Science Park, Comenius University, Bratislava, Slovakia

**Keywords:** Balance, Force plate, Mild cognitive impairment, Upright stance, Visually provoked imbalance, Visual stimulation, Sensory reweighing, Sensory conflict, Cognition

## Abstract

**Background:**

The ability to maintain balance in an upright stance gradually worsens with age and is even more difficult for patients with cognitive disorders. Cognitive impairment plays a probable role in the worsening of stability. The purpose of this study was to expose subjects with mild cognitive impairment (MCI) and healthy, age-matched controls to moving visual scenes in order to examine their postural adaptation abilities.

**Methods:**

We observed postural responses to moving visual stimulation while subjects stood on a force platform. The visual disturbance was created by interposing a moving picture in four directions (forward, backward, right, and left). The pre-stimulus (a static scene for 10 s), stimulus (a dynamic visual scene for 20 seconds) and post-stimulus (a static scene for 20 seconds) periods were evaluated. We separately analyzed the total path (TP) of the center of pressure (COP) and the root mean square (RMS) of the COP displacement in all four directions.

**Results:**

We found differences in the TP of the COP during the post-stimulus period for all stimulus directions except in motion towards the subject (left *p* = 0.006, right *p* = 0.004, and away from the subject *p* = 0.009). Significant RMS differences between groups were also observed during the post-stimulus period in all directions except when directed towards the subject (left *p* = 0.002, right *p* = 0.007, and away from the subject *p* = 0.014).

**Conclusion:**

Exposing subjects to a moving visual scene induced greater destabilization in MCI subjects compared to healthy elderly controls. Surprisingly, the moving visual scene also induced significant aftereffects in the MCI group. Our findings indicate that the MCI group had diminished adaptation to the dynamic visual scene and recovery. These results suggest that even mild cognitive deficits can impair sensory information integration and alter the sensory re-weighing process.

## Introduction

The ability to maintain an upright stance is an essential prerequisite for carrying out many activities of daily living (ADLs). The postural system involves multiple sensory inputs (proprioceptive, visual, and vestibular), an executive (motor) system, and integrative central regulation by a complex of neural systems (Horak & Macpherson, 1996). However, like many other physiological systems, the effectiveness of the postural control system decreases with age (Sturnieks, George & Lord, 2008) and it is still not fully understood which factors play decisive roles in its deterioration. Postural control relies on accurate sensory information to determine how the body is moving and maintains stability in a dynamic environment. Deficits in sensory system structure and function are important factors in the worsening of balance among the elderly (Härlein et al., 2009). Posturographic analyses during quiet bipedal stance show that sway amplitudes and velocities are greater in healthy elderly subjects compared to young people (Abrahamova & Hlavacka, 2008). Increased sway parameters are also associated with a greater number of falls, which are a major cause of morbidity (Johansson et al., 2017).

Cognitive impairment may also contribute to the deterioration of postural control. An increased risk of falling has been observed in cognitively impaired elderly subjects (Bouwen, Lepeleire & Buntinx, 2008). When sensory (vestibular, proprioceptive, and visual) compensation is needed, cognitive involvement plays a pertinent role (Horak & Kuo, 2000). Sensory adjustments are essential for everyday multitasking that demands postural instability signaling. The connection between cognition and postural control has been supported by dual-task experiments. Elderly subjects tasked with counting backwards while speaking aloud had significantly disturbed quiet stances (Swanenburg et al., 2009). Single digit naming (a working memory task) disturbed the upright stance of elderly subjects, especially those with Alzheimer’s disease (AD), both on stable and moving platforms (Rapp, Krampe & Baltes, 2006).

Many studies have used static posturography to examine the link between postural control and cognitive impairment. A systematic review published by Bahureksa et al. (2017) showed that mild cognitive impairment (MCI) had a significant effect on static postural control, both in the medio-lateral (ML) and antero-posterior (AP) sway positions with the eyes open, but not with the eyes closed. Furthermore, AP sway velocities showed greater fluctuations than ML sway velocities in MCI subjects. Leandri et al. (2009) reported decreased balance parameters in amnestic MCI patients compared to age-matched controls, and also identified AP sway as the most sensitive parameter when discriminating between healthy controls and MCI subjects (Leandri et al., 2009).

Static posturography may not be the most efficient method to detect subtle changes associated with MCI-induced postural control impairment. However, the combination of static posturography and sensory input manipulation may shed light on more complex deficits. Visual scenes moving in specific patterns can induce the illusion of body movement in the environment. Without other inputs, subjects cannot distinguish whether they are moving (self-motion) or the environment is moving (Keshner, Dokka & Kenyon, 2006). This creates a sensory conflict that demands the subject use additional cognitive resources, such as adapting their body sway to changes in the environment (Assländer & Peterka, 2014). The perception of moving visual information (and the resulting sway) may be influenced by various visual stimulation and directional characteristics such as speed (Guerraz & Bronstein, 2008) and movement (Lestienne, Soechting & Berthoz, 1977; Van Asten, Gielen & Denier van der Gon, 1988).

Sensory-motor integration, the term for the sensory-motor coupling that occurs between visual information and body sway when maintaining an upright stance, gradually decreases with age (Prioli, Freitas Júnior & Barela, 2005). A previous study involving visual feedback manipulation showed larger postural sway increases in older participants, indicating that older subjects prioritized visual input during postural control (Yeh, Cluff & Balasubramaniam, 2014). Systematic research that focuses on visual stimulation in the context of postural stability and MCI patients is scarce. The majority of related studies assessed the role of vision in postural control with opened/closed eyes, or the role of visual feedback in MCI patients, but only employed two types of visual stimulation (Szczepańska-Gieracha, Chamela-Bilińska & Kuczyński, 2012; Borges et al., 2016).

Our study investigated the influence of moving visual scenes on postural adaptation in healthy elderly controls and subjects with MCI. We hypothesized that MCI patients would show pronounced destabilization, demonstrated by increases in the total path (TP) of the center of pressure (COP), the root mean square (RMS) of the COP, and posture stabilization time.

## Materials & Methods

### Subjects

All participants were recruited from the Second Department of Neurology, University Hospital in Bratislava, Slovakia.

The MCI patient group consisted of 10 participants (five males and five females) with a mean age of 74.4 years (standard deviation [SD] = 6.6) and Montreal Cognitive Assessment (MoCA) scores between 20 and 24 points, with a mean score of 21.9 (SD = 1.6). The control group consisted of 10 (six male and four female) age-matched subjects with a mean age of 72.2 years (SD = 5.2). Their MoCA scores were between 26 and 30 points with a mean score of 27.8 points (SD = 1.7).

The inclusion criteria for the MCI group were: age ( ≥65), good visual acuity (with or without correction), and an MCI diagnosis based on the MoCA results (Nasreddine et al., 2005) and ADLs. We considered scores <25 as an indication of cognitive impairment. ADLs were assessed by standard clinical interview. Patients with severe ADL impairment were excluded. We examined their magnetic resonance imaging (MRI) scans for typical signs of microvascular leukoencephalopathy, and each subject’s scan had to be Fazekas stage 2 or higher (Fazekas et al., 1987; Fazekas et al., 1993). The MRI scans were thoroughly examined to rule out signs typical of early AD, namely significant and localized (especially posterior) cortical atrophy (Pini et al., 2016) or hippocampal atrophy (Fox et al., 1996). The inclusion criteria for the control group were: age ≥65, good visual acuity (with or without correction), no history of somatic or psychiatric disease, and no history of balance disorders. MoCA testing was also conducted for all control participants, who were required to have scores ≥25. All healthy controls underwent brain imaging (computed tomography and/or MRI) to rule out any structural changes. The exclusion criterion for both groups was any disease that may interfere with or reduce the ability to maintain balance (subjects were asked specific questions about neuropathy history, sensitivity disorders, movement disorders, and vestibular impairment).

### Ethical approval

The Ethics Committee of the University Hospital of Bratislava approved the study procedures (2015/69-UNB). Participants received comprehensive information about the experiments and gave written informed consent to participate, in accordance with the Declaration of Helsinki. The experiments were carried out in accordance with the World Medical Association’s Code of Ethics.

### Experimental setup

We adapted a standard posturographic lab by reducing ambient lighting and peripheral field of view interference when constructing the experiment room. The subjects’ peripheral field of view was reduced by curtains on both sides of the screen (to prevent visual cueing). The measuring equipment consisted of a force plate, data acquisition card, computer, projector, and a screen for back projection. We created a custom MATLAB environment for the entire acquisition process. A detailed explanation of the apparatus and calculations can be found in our earlier study (Pucik et al., 2012). The force plate was developed by the Institute of Normal and Pathological Physiology, Slovak Academy of Sciences (Hlavacka et al., 1990) and produced two analog signals proportional to the COP deviations in the ML and AP directions. The measured subject stood barefoot in a standard foot posture (forming a “V” shape with their feet at a ∼30° angle) on the force platform (Scoppa et al., 2013) in front of the projection screen. The dimensions of the projection screen were 2.0 × 1.5 m. We set the distance between the eyes and screen at 0.75 m.

### Visual stimuli

There is currently no “gold standard” for visual motion stimuli design, but various test patterns and movements have been reported. Previous research on visual stimulation usually employed sinusoidal stimuli (Peterka & Benolken, 1995; Mergner et al., 2005; Dokka et al., 2010). Other known stimulation scenes rotated an image around the horizontal plane (Day et al., 2016), or created and tested multiple scenes that closely resembled situations that could provoke postural disturbance (Pucik et al., 2014). However, these scenes did not elicit the expected response. In this study, we used high contrast scenes because they showed greater potential for inducing postural sway and imbalance.

**Figure 1:**
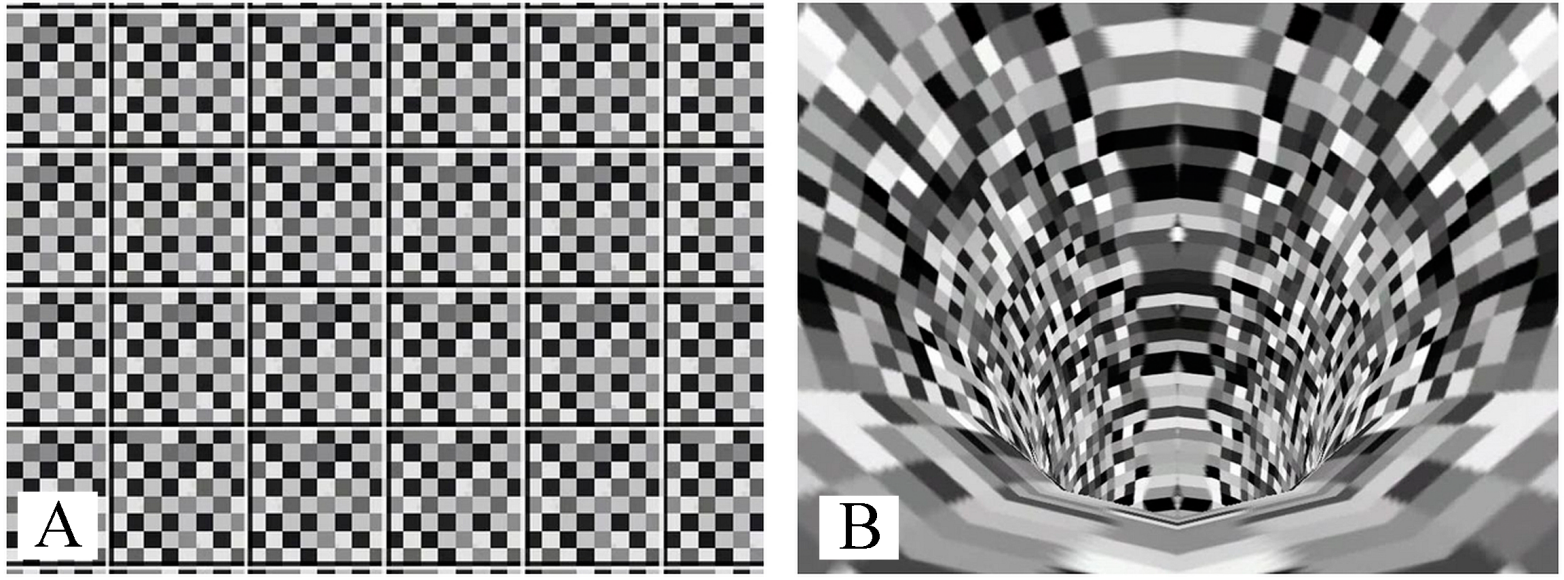
Images used for ML stimulation (A) and AP stimulation (B).

Using a Virtual Reality Modeling Language format, we rendered visual stimuli as 3D objects moving in time. The scene used for the illusion of lateral movement (a “roll” movement along the *x*-axis) consisted of a checkered board that rotated around an axis aligned with the approximate center of the measured subject’s body mass (see Fig. 1A). The checkered pattern surface was chosen to eliminate “cueing” of vision on any solid object in the visual field. For the illusion of forward and backward motion along the *y*-axis, we used a scene with an animated tube (see Fig. 1B). The checkered pattern surface was used for the same reason as in the lateral scene. Both scene types moved at a constant angular velocity. The ML scene rotated at an angular velocity of 36°/s. This rotation produced linear velocity with a horizontal component of 1.1 m/s in the central field of view, and the velocity vector magnitude increased up to 1.6 m/s in the peripheral field of view. In the AP scene (Fig. 1B), image points in the center moved vertically at 0.57 m/s, while the optical flow was 2.5 m/s in the periphery. We used the MATLAB environment to play and stop the files during the experiments.

We analyzed the signals on a 100-Hz frequency after 12-bit AD conversion, and filtered them using a zero-phase fourth-order Butterworth filter with a cut-off frequency of 20 Hz. Measured COP positions were used to calculate the TP of the COP, RMS in the anteroposterior (RMS_AP_), and RMS in the mediolateral (RMS_ML_) directions. Standard equations were used to calculate the TP of the COP and RMS, and are explained in detail in our previous study (Pucik et al., 2012).

### Protocol

Each participant underwent the same measurement protocol for their responses to scenes in four directions (right, left, away from the subject, and towards the subject). Each response was measured for a duration of 50 s, consisting of the 10-second pre-stimulus period (static scene), followed by the 20-second stimulation period (moving scene), and the 20-second post-stimulus period (static scene). Each scene was presented five times in a pseudo-random order (the directions were randomly shuffled to suppress adaptation). A total of 20 scenes were presented. After every two scenes, there was a break for at least 120 s to decrease the influence of the adaptation effect and to prevent fatigue.

### Computer and statistical analyses

We evaluated postural stability using the TP of the COP, the RMS_ML_, and the RMS_AP_. The TP of the COP is a representative velocity-related measure that provides information on the postural corrections required to maintain postural stability, while the RMS reflects displacement-related measures that allow for the estimation of overall standing postural performance (Hufschmidt et al., 1980; Holliday & Fernie, 1990).

The descriptive statistics were separately calculated for each phase (pre-stimulus, 10 s; stimulus, 20 s; and post-stimulus, 20 s). We also separately analyzed the measurements across the four different directions (away from, towards, left, and right). The three phases (pre-stimulus, stimulus, and post-stimulus) were compared within groups and across different groups. We used IBM SPSS version 24.0 software for statistical analyses.

Repeated measures analyses of variance (ANOVAs) were utilized to calculate the TP of COP and RMS scores. We tested two types of models. In the first model, we considered stimulus type and direction as within-group factors and group as a between-subject factor. Four simpler models were also tested in all directions, with stimulus type as a within-group factor and group as a between-subject factor. We used a Greenhouse-Geisser correction when a violation of sphericity occurred. For post-hoc analyses, *t*-tests were used. When groups were compared across stimulus type, we performed a Bonferroni correction (alpha_bonf_ = alpha/number of comparison). Group differences were considered statistically significant when *p* ≤ 0.0167. The effect sizes were expressed with η^2^ coefficient (we used following cut offs for quantification of size: small - η^2^ = 0.01; medium - η^2^ = 0.06; and large - η^2^ = 0.14) (Miles & Shevlin, 2001).

## Results

**Figure 2:**
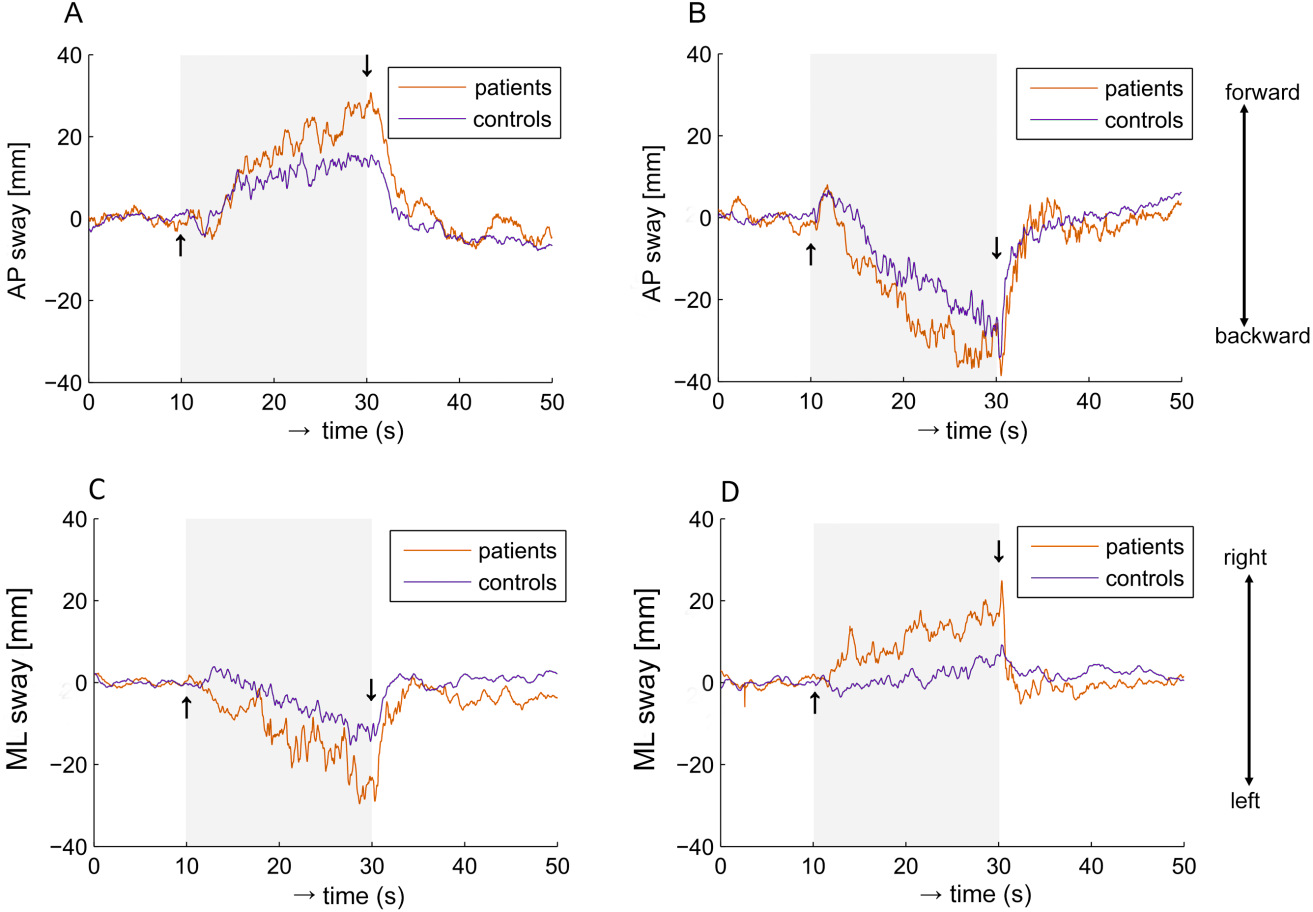
Averaged group responses of COP shift to visual stimulation. (A) Scene moving forward from the subject. (B) Scene moving backwards from the subject. On the vertical axis, values above and below zero indicate the subject moving on AP axis forward and backward. (C) Scene rotating to the left. (D) Scene rotating to the right. On the vertical axis, values above and below zero indicate the subject is moving on ML axis to the right and left, respectively.

We found that a moving visual field induced responses in the same direction for both the MCI and control groups. Figure 2 shows the postural sway elicited by visual stimulation.

**Figure 3:**
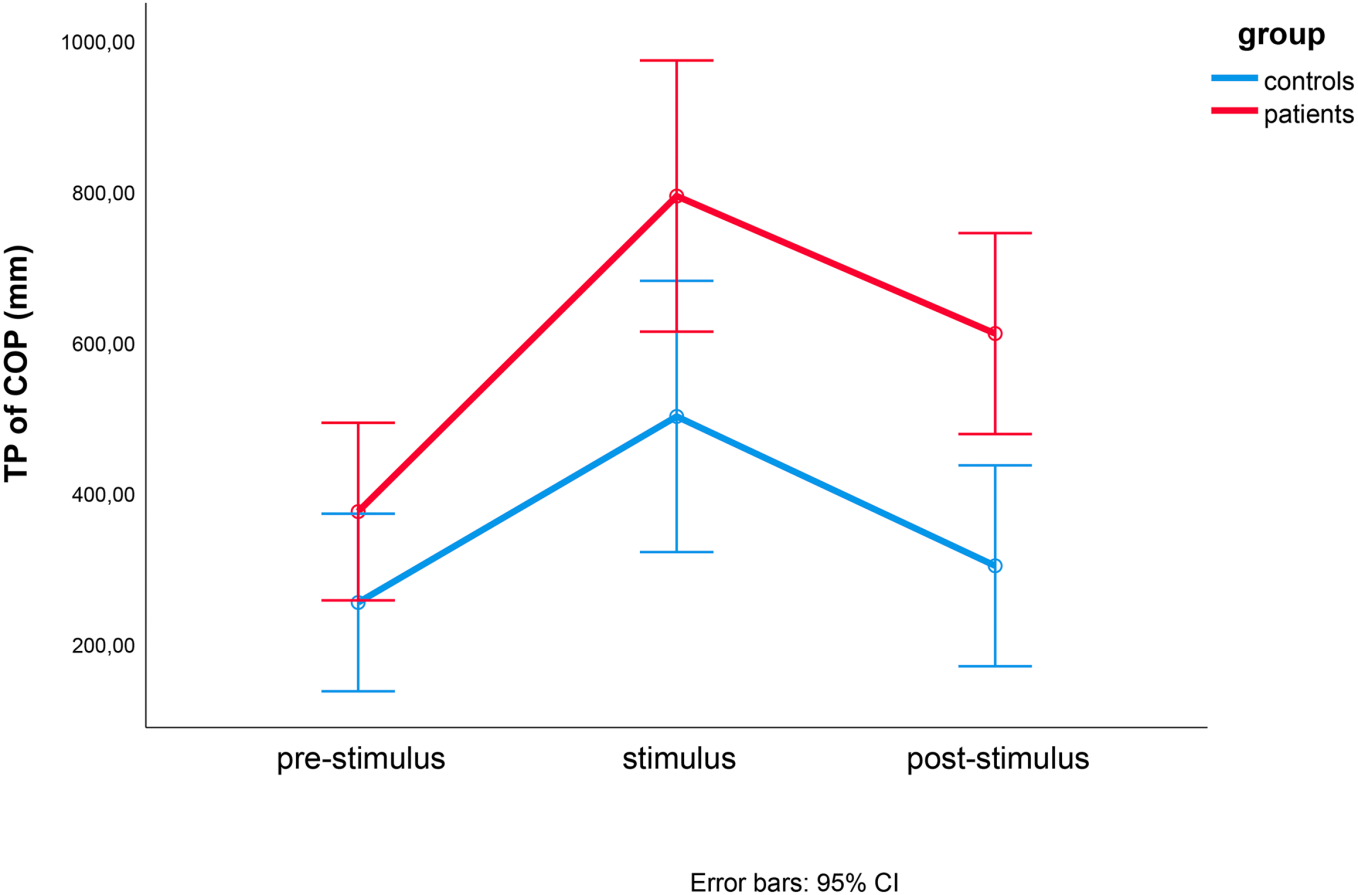
Results of repeated measures ANOVAs of TP of COP. Repeated measures ANOVAs of TP of COP between controls (blue) and MCI patients (red) during the pre-stimulus, stimulus and post-stimulus periods. Time (pre-stimulation, stimulation and post-stimulation) and group were within- and between-factor variables, respectively.

**Figure 4:**
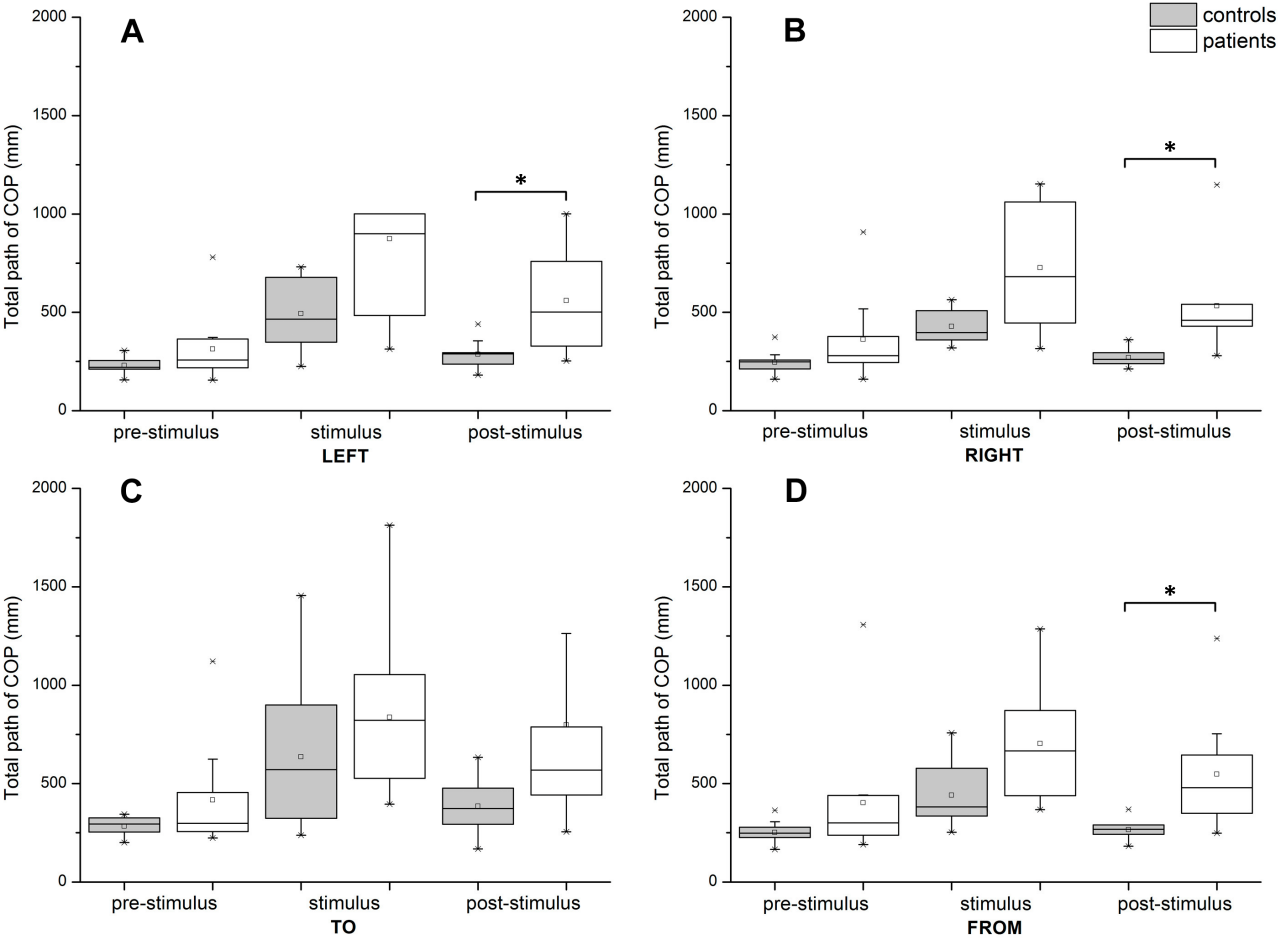
Post-hoc *t*-tests comparing TP of COP between groups during pre-stimulus, stimulus, and post-stimulus periods. (A) Scene rotating to the left. (B) Scene rotating to the right. (C) Scene moving to subject. (D) Scene moving away from the subject. Significant differences (*p* ≤ 0.0167) are marked with asterisks. In post-stimulus period, for direction to the left *p* = 0.009, for right *p* = 0.007 and for direction from the subject *p* = 0.012.

In the TP of the COP, we observed significant effects caused by time (*F*(1.316, 23.683) = 26.803, *p* < 0.001, eta^2^ = 0.598), direction (*F*(1.572, 28.302) = 3.877, *p* = 0.042, eta^2^ = 0.177), and group (*F*(1, 18) = 8.379, *p* = 0.010, eta^2^ = 0.318). This three-way interaction effect was not statistically significant (*F*(1.887, 33.972) = 1.206, *p* = 0.310, eta^2^ = 0.063) (Fig. 3). The post-hoc *t*-test results are shown in Fig. 4.

**Figure 5:**
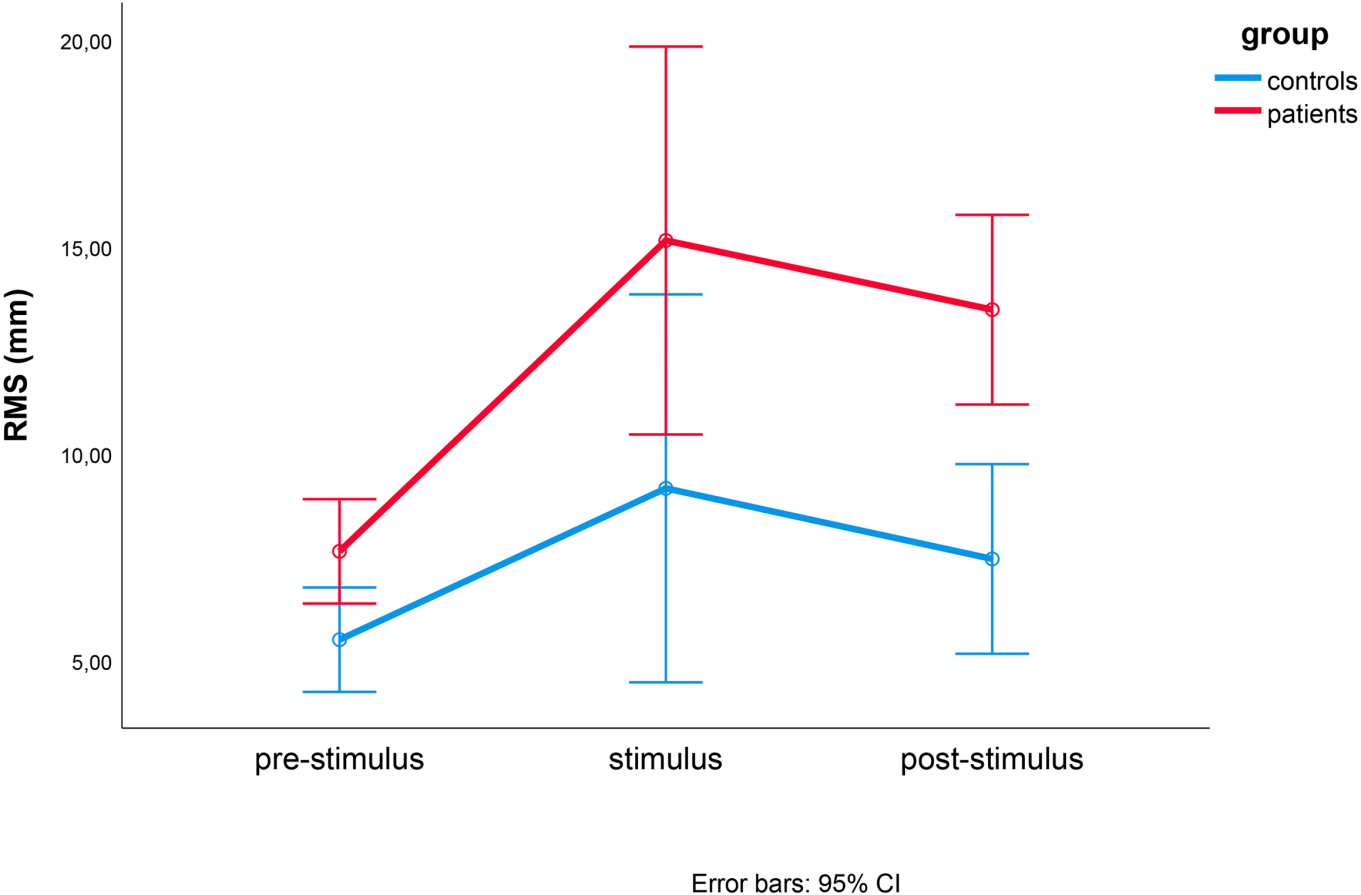
Results of repeated measures ANOVA of RMS differences between controls (blue) and MCI patients (red) during pre-stimulus, stimulus, and post-stimulus periods. Time (pre-stimulation, stimulation and post-stimulation) and group were within- and between-factor variables, respectively.

**Figure 6:**
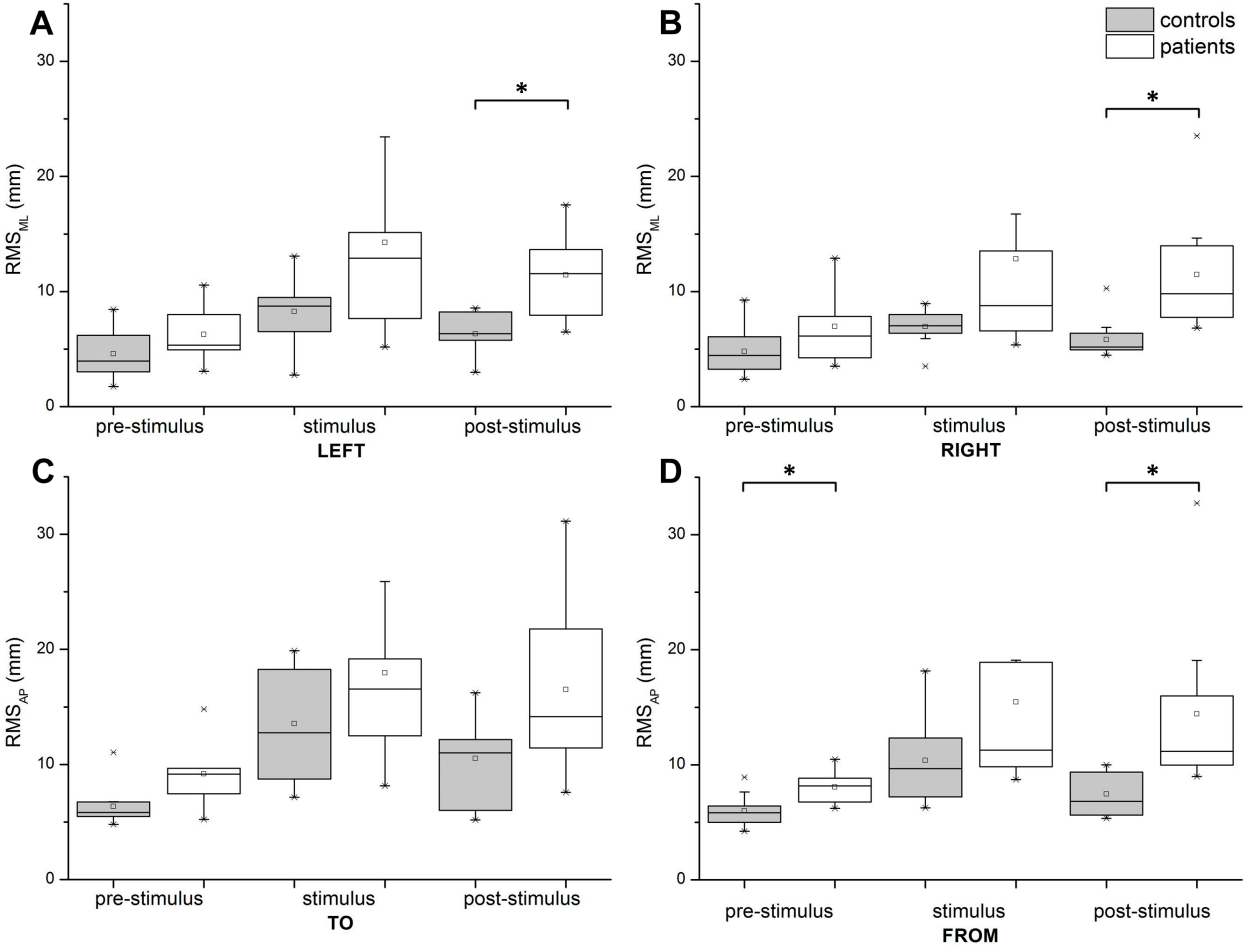
Post-hoc *t*-tests comparing RMS (in mm) between groups during pre-stimulus, stimulus, and post-stimulus periods. (A) Scene rotating to the left. (B) Scene rotating to the right. (C) Scene moving to subject. (D) Scene moving away from the subject. Significant differences (*p* ≤ 0.0167) are marked with asterisks. In pre-stimulus period, for stimulation away from subject, *p* = 0.003. In post-stimulus period, for direction to the left *p* = 0.009, for right *p* = 0.007 and for direction away from the subject *p* = 0.014.

For the RMS, we observed significant effects caused by time (*F*(1.177, 21.183) = 12.869, *p* = 0.001, eta^2^ = 0.417), direction (*F*(2.074, 37.340) = 14.821 *p* < 0.001, eta^2^ = 0.482), and group (F(1, 18)=8.388, *p* = 0.010, eta^2^ = 0.318). This three-way interaction effect was not statistically significant (*F*(3.068, 55.229) = 0.357, *p* = 0.789, eta^2^ = 0.019) (Fig. 5). The comparisons between the RMS_ML_ for lateral stimulation and the RMS_AP_for AP stimulation (post-hoc *t*-test) in all directions are shown in Fig. 6.

The separate repeated measures ANOVA results and the descriptive statistics for the TP of the COP and RMS for the four directions are presented in Table 1. In these models, time (pre-stimulation, stimulation, and post-stimulation) was the within-group factor and group was the between-subject factor. We found significant differences across different phases (pre-stimulation, stimulation, post-stimulation) except for the RMS when stimulating to the right side. In group comparison (healthy controls and MCI patients) we found significant differences in all conditions, but not in TP of the COP when stimulating toward the subject. The interaction between stimulus and group was not significant neither in the TP of the COP nor in RMS in all directions.

## Discussion

Both groups responded to the dynamic visual scenes with postural tilts and new COP positions. The postural tilts were in the same rotating and linear directions of motion shown in the given scene. These observations were consistent with those reported previously for postural responses perturbed by moving visual stimuli (Meyer et al., 2013). We did not find any differences in the postural reactions of the control and MCI groups during stimulation, but we did observe a difference after the cessation of the visual stimulus during the post-stimulus period. This difference was reflected in both the TP of the COP and RMS values in the directions to the left, right, and away from the subject.

**Table 1:** Results of separate repeated measures ANOVAs of TP of COP and RMS.^a^

		**Pre-stimulus**		**Stimulus**		**Post-stimulus**		**Repeated measures ANOVA**		
		**controls**	**patients**	**controls**	**patients**	**controls**	**patients**	**TIME STIMULATION PHASE**	**GROUP**	**INTERACTION**
		group averages in milimeters (SD in brackets)						****	****	****
**TP of COP**	**LEFT**	230.1 (43.2)	312.7 (178.6)	493.6 (165.2)	900.2 (542.3)	286.3 (71.3)	559.1 (260.8)	*F* = 17.177, p <0.001, η^2^ = 0.488	*F* = 8.546, *p* = 0.009, η^2^ = 0.322	*F* = 1.589, *p* = 0.144, η^2^ = 0.111
****	**RIGHT**	245.7 (57.1)	361.6 (216.5)	428.2 (88.2)	726.6 (333.4)	269.3 (44.9)	532.9 (245.1)	*F* = 21.372, p <0.001, η^2^ = 0.543	*F* = 9.626, *p* = 0.006, η^2^ = 0.348	*F* = 2.609, *p* = 0.112, η^2^ = 0.100
****	**TO**	283.5 (49.3)	416.8 (275.3)	635.6 (386.0)	836.9 (425.5)	385.1 (143.8)	798.7 (642.3)	*F* = 9.309, *p* = 0.001, η^2^ = 0.341	*F* = 3.628, *p* = 0.073, η^2^ = 0.168	*F* = 1.306, *p* = 0.284, η^2^ = 0.068
****	**FROM**	251.9 (58.0)	402.6 (328.4)	441.0 (172.2)	703.8 (302.9)	265.5 (49.4)	547.5 (286.1)	*F* = 29.667, p <0.001, η^2^ = 0.622	*F* = 5.869, *p* = 0.026, η^2^ = 0.246	*F* = 1.457, *p* = 0.124, η^2^ = 0.117
**RMS**	**LEFT**	4.82 (2.13)	6.28 (2.55)	7.83 (2.72)	14.26 (9.82)	6.48 (2.22)	11.43 (3.56)	*F* = 9.381, *p* = 0.004, η^2^ = 0.343	*F* = 8.225, *p* = 0.010, η^2^ = 0.314	*F* = 1.988, *p* = 0.172, η^2^ = 0.099
****	**RIGHT**	4.93 (2.10)	6.96 (3.34)	6.87 (1.50)	12.83 (12.29)	5.90 (1.71)	11.48 (5.05)	*F* = 3.387, p <0.076, η^2^ = 0.554	*F* = 5.993, *p* = 0.025, η^2^ = 0.250	*F* = 0.991, *p* = 0.343, η^2^ = 0.052
****	**TO**	6.31 (1.79)	9.17 (2.92)	12.53 (4.63)	17.96 (8.95)	9.94 (3.28)	16.51 (7.67)	*F* = 18.896, p <0.001, η^2^ = 0.512	*F* = 6.183, *p* = 0.023, η^2^ = 0.256	*F* = 1.129, *p* = 0.335, η^2^ = 0.059
****	**FROM**	5.87 (1.43)	8.06 (1.44)	9.33 (2.53)	15.47 (9.98)	7.42 (1.82)	14.41 (7.21)	*F* = 10.880, p < 0.001, η^2^ = 0.377	*F* = 7.293, *p* = 0.015, η^2^ = 0.288	*F* = 2.226, *p* = 0.140, η^2^ = 0.112

**Notes.**

a Descriptive values are given with standard deviations in parentheses.

The patients remained unstable during the whole recorded (post-stimulus) period, i.e., the COP position did not reach the initial set. One reason for this stimulation after-effect could be the persistent feeling of self-motion (vection). Guerraz & Bronstein (2008) proposed two different postural reactions that differed in latency and origin. The longer latency postural mechanism was related to the conscious perception of self-motion during longer-duration body displacements, and the second system could be vection-influenced. This system takes time to fully develop and consistently induce body tilt in the direction of visual motion. Based on the duration of the stimulation, a longer latency system could “kick in” during movement cessation or even later, increasing sway during the post-stimulus period.

Vection is perceived differently in subjects with MCI, since cognitive status is known to alter the recognition of vection (Riecke et al., 2006). Mather et al. (2008) summarized that adaptation to visual motion perception (and the experience of motion after-effect) involves up to five different cortical areas, reflecting the multiple levels of processing involved in visual motion analysis. Healthy but fall-prone seniors need more time to adapt to changes in visual stimulus amplitude compared to young people (Jeka, Allison & Kiemel, 2010). Furthermore, healthy seniors need more exposure to visual motion perturbation in order to habituate themselves, and this may be exacerbated in MCI patients (O’Connor & Kuo, 2009). As a result, subjects with MCI may have more difficulties mastering vection and the consequent postural responses needed to respond to changes in static and dynamic scenes.

Studies on augmented visual feedback have found that elderly adults and subjects with MCI prioritize vision during postural control (Szczepańska-Gieracha, Chamela-Bilińska & Kuczyński, 2012; Yeh, Cluff & Balasubramaniam, 2014). This reflects a compromised ability to correctly reweigh visual information and an overreliance on visual input. The MCI group’s persistent postural destabilization after visual stimulation cessation may have been caused by their loss of ability to quickly downweigh the importance of visual information during postural stabilisation. This top-down inhibition of sensory information may be mediated by the cholinergic system, which is deficient in patients with subcortical vascular lesions associated with MCI (Murray et al., 2018; Liu et al., 2017). We also observed similar difficulties with downweighing visual information and fusing different sensory modalities when adaptation was needed in subjects with Parkinson’s disease (PD), which is associated with subcortical cognitive impairment (Hwang et al., 2016). PD patients can also react hyperactively to visual stimuli, and can find visual information to be misleading (Bronstein et al., 1990).

Despite extensive efforts, we did not find any relevant studies describing motion after-effects in subjects with MCI or any cognitive disorder of subcortical vascular origin. However, multiple studies have observed akinetopsia (the inability to distinguish movement in a visual scene) in subjects with AD (Tsai & Mendez, 2009). Our results did not rule out the possibility that subjects with MCI of a vascular origin (as opposed to subjects with AD) may sway more in a variable environment due to their increased sensitivity to motion after-effect.

Compensatory postural feedback mechanisms are impaired in people with white matter lesions (Zheng et al., 2011). In our MCI group, we observed increased sway parameters during the post-stimulus phase that suggest alterations in the feedback mechanisms. The persistent instability of the MCI group during the post-stimulus phase supports the assumption that these subjects have a decreased ability to adapt their perception to a dynamic visual scene.

The non-significant results for the TP of the COP and RMS _*AP*_ between the MCI and control groups in the direction towards the subject (both in the stimulus and post-stimulus periods) may be explained by the biomechanical restrictions on backward ankle joint movement and the shorter distance towards safe posture boundaries (Horak, 1997). Another explanation could be the subjects’ fear of falling, which may have activated their stiffening strategies and smaller backward postural reactions (Carpenter et al., 2001).

### Study limitations

The sample size of our studied population was small, but the statistical significance of the results and the thorough age matching between groups supports their validity. A statistical RMS_AP_ difference could have affected the RMS_AP_ values during and after stimulation. However, a statistical difference was only achieved during the post-stimulus period. Our findings are also in line with those of Novak et al. (2009), who found increased sway parameters in patients with periventricular white matter lesions. Additionally, we only performed force-plate measurements, and more accurate data may have been obtained using an accelerometer and a 3D tracking system. The test groups included almost equal numbers of males and females, and we did not address gender differences in terms of postural sway. We also could not rule out subjects in the early stages of other forms of cognitive dysfunction (but with the same MRI and clinical characteristics as subjects with vascular-origin MCI) using our methods. Knowing the prevalence of previous and, more importantly, future falls could add key information to the clinical application of our findings.

## Conclusion

In conclusion, we examined how a visual dynamic scene influences postural stability in elderly subjects with MCI and age-matched controls. We found that the dynamic visual scene caused MCI patients to have more pronounced postural reactions that allowed them to maintain postural stability within safe boundaries. The most striking difference between the healthy controls and the MCI patients occurred during the post-stimulus period after the cessation of the visual stimulation. There are several possible explanations for this phenomenon. The most likely is the subjects’ decreased abilities to habituate to aberrant visual perception and to rapidly assess sensory information source reliability. This means that patients were more dependent on dominant sensory information, even when it was incorrect. Therefore, continuous sensory illusions can cause destabilisation and even falls in MCI patients.

##  Supplemental Information

10.7717/peerj.10363/supp-1 Supplemental Information 1 Raw data of all subjects (Total path of COP in mm)

10.7717/peerj.10363/supp-2 Supplemental Information 2 Raw data of all subjects (RMS of Center of Pressure in mm)

10.7717/peerj.10363/supp-3 Supplemental Information 3 Results of separate repeated measures ANOVAs of TP of COP and RMS^a^ ^a^ Descriptive values are given with standard deviations in parentheses.

10.7717/peerj.10363/supp-4 Supplemental Information 4 Clip presenting visual stimulation in medio-lateral direction (rotation to the left/right)

10.7717/peerj.10363/supp-5 Supplemental Information 5 Clip presenting visual stimulation in antero-posterior direction (endless tube moving forward/backward)

## Additional Information and Declarations

### Data Availability

The following information was supplied regarding data availability: The raw measurements are available as Supplemental File.
